# Evaluation of the impact of different drying methods on the phenolic compounds, antioxidant activity, and in vitro digestion of green coffee beans

**DOI:** 10.1002/fsn3.948

**Published:** 2019-02-11

**Authors:** Ke Cheng, Wenjiang Dong, Yuzhou Long, Jianping Zhao, Rongsuo Hu, Yanjun Zhang, Kexue Zhu

**Affiliations:** ^1^ Spice and Beverage Research Institute Chinese Academy of Tropical Agricultural Sciences Wanning China; ^2^ College of Food Science and Technology Huazhong Agricultural University Wuhan China; ^3^ Tropical Crops Genetic Resources Institute Chinese Academy of Tropical Agricultural Sciences Danzhou China

**Keywords:** antioxidant activity, coffee beans, drying methods, in vitro digestion, phenolics

## Abstract

We investigated the effects of different drying methods (room temperature drying, heat pump drying, freeze drying, microwave vacuum drying [MVD], and combined microwave power vacuum drying) on the active ingredients of green coffee beans. We specifically focused on eleven phenolic acids, total phenolic content (TPC), total flavonoid content, antioxidant activity, polyphenol oxidase (PPO), the inhibition of linoleic acid peroxidation (LPO), and enzyme activity, and the bio‐accessibility in vitro and bioavailability of phenolics and antioxidant activities were also evaluated. MVD‐treated beans had the lowest PPO activity and the highest contents of 5‐caffeoylquinic acid (1.39 g/100 g DW), 3‐feruloylquinic acid (0.25 g/100 g DW), 4‐feruloylquinic acid (0.25 g/100 g DW), 5‐feruloylquinic acid (1.52 g/100 g DW), and TPC (5.98 g GAE/100 g DW), and the highest antioxidant activity. MVD was the least time‐consuming (0.63 hr/kg) and energy‐consuming (1.88 kwh/kg) method. Moreover, the strongest inhibition of LPO and α‐glucosidase was observed in MVD‐treated beans. Thus, MVD is suggested to be the most suitable drying technique for the preservation of phenolic compounds and bioactivities of green coffee beans.

## INTRODUCTION

Coffee is a shrub that belongs to the family Rubiaceae, genus *Coffea*. It is widely cultured in tropical areas, and in China, it is mainly distributed in the Hainan and Yunnan provinces (Dong, Tan, Zhao, Hu, & Lu, 2015). Robusta (*Coffea canephora* var. *robusta*) and Arabica (*Coffea arabica*) are the two varieties dominating the international market. The global production of coffee cherry has been estimated at 8 million metric tons per year, which shows its economic importance (Battista, Fino, & Mancini, 2016). Recently, critical epidemiological and intervention studies have shown that moderate daily consumption of coffee has various positive effects on the human body (O'Keefe et al., 2013). Coffee intake may offer protection against cardiovascular disease, type 2 diabetes, coronary heart disease, stroke, depression, and neurological diseases, including Parkinson's and Alzheimer's diseases (Cano‐Marquina, Tarín, & Cano, 2013). Substantial evidence indicates that these health‐promoting properties of coffee are linked to its phenolics, especially chlorogenic acids (Gómez‐Ruiz, Leake, & Ames, 2007). Furthermore, coffee beans are rich in antioxidants and nutrients (Fukushima et al., 2009).

Phenolics possess antioxidant, anti‐inflammatory, and anticancer activities, and are widely used for food fortification (Arts & Hollman, 2005; Kamiloglu et al., 2016; Wang, Melnyk, Tsao, & Marcone, 2011). Raw coffee brew is widely used as a functional additive owing to its high content of active ingredients. Świeca, Gawlik‐Dziki, Dziki, and Baraniak (2017) used raw coffee brew as a functional fortification enriched in wheat bread, Dziki et al. (2015). reported the use of green coffee beans as a functional additive. Generally, high activity in vitro does not translate to high activity in vivo;(González‐Sarrías, Tomé‐Carneiro, Bellesia, Tomás‐Barberán, & Espín, 2015; Siviero et al., 2015) thus, it is necessary to determine the bio‐accessibility and bioavailability of functional supplements in some new products. However, in vivo studies are usually difficult to perform and very expensive. Therefore, some in vitro methods based on simulated digestion and absorption have been introduced and successfully exploited (Hur, Lim, Decker, & McClements, 2011).

Coffee cherries are susceptible to rotting and fungal infection, which have a substantial impact on the quality of coffee. The timely dehydration of ripe coffee cherries can effectively prolong their storage time and contribute to their usability in food production (Knopp, Bytof, & Selmar, 2006). Traditionally, coffee cherries are dried in sunlight or shade, which is widely used by farmers and in factories. However, during long‐time drying, problems such as mustiness can arise. The current main industrial methods for fruit and vegetable drying include heat pump drying, freeze drying, and microwave vacuum drying. Heat pump drying has been widely applied in agricultural product processing because of the relatively low equipment cost. Freeze drying has been reported to be the best drying method for maintaining maximum levels of active components in fruits and vegetables, but the expensive equipment limits large‐scale application. Microwave vacuum drying is a new, fast, and economic drying method that can prevent deterioration of agricultural products (Fan et al., 2012). Chang, Lin, Chang, and Liu (2006) reported that dried tomatoes have higher phenolic contents than fresh ones, whereas Rodríguez et al. (2014) found opposite results in murta berries. Thus, drying processes may have positive or negative effects on active ingredients in coffee beans compared to green coffee beans.

The increasing prevalence of obesity and type 2 diabetes is associated with caloric intake and diet composition. The inhibition of nutrient digestion and absorption is one of the effective strategies to prevent obesity and type 2 diabetes. In previous studies, inhibition of the activity of pancreatic lipase, α‐glucosidase, and α‐amylase, which are involved in dietary carbohydrate and lipid degradation, has been found to be positively correlated with reduced fat and sugar absorption from the gastrointestinal tract (Fu, Jiang, Guo, & Su, 2016; Kumar, Narwal, Kumar, & Prakash, 2011; Sales, Souza, Simeoni, Magalhães, & Silveira, 2012). It has been reported that modest coffee intake can slow down diabetes. To the best of our knowledge, no studies have been conducted to investigate the comparative analysis of different drying procedures on the phenolic compounds, antioxidant activities, and in vitro digestion of green coffee beans.

In the current study, we determined the effects of four drying processes (room temperature drying, heat pump drying, freeze drying, microwave vacuum drying, and combined microwave power vacuum drying) on (a) the contents of eleven phenolic acids; (b) in vitro bio‐accessibility and bioavailability of phenolics and antioxidants; (c) digestive enzyme inhibition; and (d) polyphenol oxidase, inhibition of linoleic acid peroxidation, and Maillard reaction products. And the aim of this study was to explore which drying method was best for preserving the bioactive components of green coffee beans, so as to providing a theoretical basis for the industrial production of functional food additives.

## EXPERIMENTAL

### Chemicals and reagents

Folin–Ciocalteu reagent, gallic acid, potato starch, linoleic acid, pancreatin (P 110505) from porcine pancreas, and α‐amylase (A 109181) from bacteria were purchased from Aladdin (Shanghai, China). Rutin, bovine hemoglobin, acarbose, and α‐glucosidase (S 10050) from yeast were obtained from Yuanye Biotechnology (Shanghai, China). Mucin, pepsin, bile extract, ammonium pyruvate, polyvinylpolypyrrolidone (PVPP), Triton X‐100, 4‐methylumbelliferyl α‐d‐glucopyranoside (4‐MUG), 4‐methylumbelliferyl oleate (4‐MUO), Tris base, orlistat, diphenyl picryl hydrazyl radical (DPPH), 2,2′‐azinobis‐(3‐ethyl‐benzothiazoline‐6‐sulfonic acid) (ABTS), 2,4,6‐tris‐(2‐pyridyl)‐S‐triazine (TPTZ), 6‐hydroxy‐2,5,7,8‐tetramethychromane‐2‐carboxylic acid (Trolox), and lipase (L 3126) from porcine pancreas type II, and the standards (all phenolic acids) were purchased from Sigma.

### Sample preparation

Coffee cherries (*Coffea canephora* var. *robusta*) were harvested in March 2017 at the Spice and Beverage Research Institute of the Chinese Academy of Tropical Agricultural Sciences in Hainan, China. All cherries were fresh, full, healthy, and nearly red, and three samples from different experimental base were used in this study. Green coffee beans were obtained by removing the peel and mucilage of coffee cherries mechanically and immediately dried.

### Drying methods

#### Microwave vacuum drying (MVD)

A multifunctional and stable microwave vacuum dryer (Type WBZ‐10PLC), with a maximum microwave power of 2.0 kW and a stable vacuum condition of −0.085 MPa, was purchased from Guizhou Xinqi Microwave Industry Co., Ltd. (Guiyang, Guizhou, China). Fresh coffee beans (2.0 kg) were placed in thin layers on two plates and put into the microwave cavity. The equipment was operated for 75, 160, and 280 min at a microwave power of 1.0, 0.5, and 0.3 kW (MVD‐1.0, MVD‐0.5, and MVD‐0.3), respectively. A combined microwave vacuum drying method (CMVD) was employed at the same power levels mentioned above (1.0 + 0.5 + 0.3 kW). The drying process (all MVD conditions) was continued until the moisture content of the coffee beans reached 11.0% on wet basis.

#### Room temperature drying (RTD)

Room temperature drying was performed in a ventilated open area at a temperature between 20 and 30°C and a relative humidity of approximately 70%. The beans (2.0 kg) were dried until the moisture content of beans reached 11.0 ± 1.0/100 g dry weight (DW).

#### Heat pump drying (HD)

The samples were dried using an electric thermal dryer as reported by Dong, Hu, Chu, Zhao, and Tan (2017). The drying temperature was set at 40 or 50°C (HD‐40, HD‐50) at an air flow rate of 1.0 m/s. The beans (2.0 kg, all HD conditions) were dried until their water content reached 11.0% (wet basis).

#### Freeze drying (FD)

Freeze drying was carried out in a freeze dryer with three drying plates (JDG‐0.2; Lanzhou Kejin Freeze Drying Instruments Co., Ltd., Lanzhou, China). Coffee beans that had been frozen at −20°C for 24 hr were placed on the plates and dried under 0 Pa vacuum pressure until the moisture content reached 11.0% (wet basis). The heating plate and cold trap temperatures were set at 50°C (FD‐50) and −40°C, respectively.

### Phenolic compound content

#### Phenolic compound extraction

Two grams of coffee powder dried by each method was extracted with 50 ml of 80% ethanol aqueous solution at 80°C for 3 hr. The extract solution was used to analyze the contents of effective compounds, namely phenolic acids, total phenolic content, DPPH, ferric reducing antioxidant power (FRAP), and ABTS. Each sample was determined in triplicate, and the mean of three measurements was used for further analysis.

#### Total phenolic content (TPC)

The TPC of coffee beans dried by the different drying methods was quantified by the Folin–Ciocalteu colorimetric method (Jayaprakasha, Selvi, & Sakariah, 2003). The absorbance at 750 nm was measured with a UV‐Vis spectrophotometer (SPECORD 250 PLUS; Analytik Jena, Jena, Germany). The TPC was expressed as gallic acid equivalents (g GAE/100 g DW). Each sample was determined in triplicate.

#### Total flavonoid content (TFC)

The TFC of coffee beans dried by the different drying methods was determined using the method of Chu, Chang, and Hsu (2000). The absorbance at 510 nm was recorded with a UV‐Vis spectrophotometer. Total flavonoid data were expressed as rutin equivalents (g rutin/100 g DW). Each sample was determined in triplicate.

#### Phenolic acid contents

Eleven phenolic acids were analyzed as follows: 3‐CQA, 4‐CQA, 5‐CQA, 3,4‐diCQA, 3,5‐diCQA, 4,5‐diCQA, 3‐FQA, 4‐FQA, 5‐FQA, 1,3,5‐triCQA, and l‐EP. For phenolic acids analysis, 0.2 ml sample extract was first filtered through a 0.22‐μm organic membrane, and then, the phenolic acid contents were determined on an Agilent UPLC 1290 system (Agilent Technologies Inc., Palo Alto, CA, USA) consisting of an automatic sample manager, a diode array detector, and a reverse‐phase C18 column (100 mm, 2.1 mm, i.d., 1.7 mm particle size). The mobile phase consisted of two components: methanol (A) and 0.1% (v/v) methanoic acid aqueous solution (B). Gradient elution conditions were as follows: 0–70 min, 5%–100% A; 70–75 min, 100%–100% A. The mobile phase flow rate was 0.4 ml/min. The column temperature was 25°C, and the injection volume was 1.5 μl. The detector was set at 330 nm. All samples were analyzed in triplicate.

### Antioxidant activity assessment

The DPPH radical scavenging assay, based on the measurement of the scavenging ability of antioxidants toward the stable radical DPPH, was conducted according to Almeida et al. (2011). The FRAP assay was performed according to the method reported by An et al. (2016). The ABTS antioxidant activity (AA) was determined following the method of Zheng, Xia, and Lu (2015). The absorbance for DPPH, FRAP, and ABTS determinations was measured at 515, 593, and 734 nm, respectively. The antioxidant ability was represented as EC_50_: the amount of sample (mg dried sample, DW) required to obtain 50% activity per 1.0 ml of the extract solution. Each sample was determined in triplicate.

#### Inhibition of linoleic acid peroxidation (LPO)

The inhibition of linoleic acid peroxidation was determined via hemoglobin‐catalyzed peroxidation (Goupy, Vulcain, Caris‐Veyrat, & Dangles, 2007). The absorbance of the sample (A_s_) was measured at 480 nm using a spectrophotometer. The absorbance of a blank (A_b_) sample, that is, the reaction mixture without the addition of hemoglobin, and of a control (A_c_) sample, that is, the reaction mixture without the addition of coffee bean extract, was obtained. Each sample was determined in triplicate. The LPO inhibition ratio of the samples was calculated according to the following equation:

(1) $$\text{LPO}\left(\%\right)=\left[1-\frac{\left(A_{S}-A_{b}\right)}{\left(A_{C}-A_{b}\right)}\right]\times 100$$

### Polyphenol oxidase (PPO) assay

The PPO activity was measured according to the method reported by Sulaiman, Soo, Yoon, Farid, and Silva (2015). Enzyme solution (50 μl) was added to 150 μl of catechol solution (0.2 M in phosphate buffer, pH 5.8). The absorbance at 410 nm was measured using a spectrophotometer. Each sample was determined in triplicate.

### Enzyme inhibition assays

#### Enzyme extraction

Samples of the coffee beans dried by the various methods (2.0 g of DW) were extracted for 2 hr with 50 ml of 0.2 M phosphate‐buffered saline (pH 7.4). The extracts were filtered, and the filtrates were stored in the dark at −20°C for further study.

#### α‐Amylase inhibition assay

The α‐amylase inhibitory effect of the different drying methods was assayed according to the procedure described by Podsedek, Majewska, Redzynia, Sosnowska, and Koziołkiewicz (2014). The absorbance at 600 nm (Synergy H1; BioTek Instruments Inc., Winooski, VT, USA) was recorded. All samples were assayed in triplicate. The α‐amylase inhibitory activity was calculated as follows:

(2) $$\text{inhibition}\left(\%\right)=\left[1-\frac{\left(A_{B}-A_{A}\right)}{\left(A_{D}-A_{C}\right)}\right]\times 100,$$

where A_A_ and A_B_ are the absorbances of mixed solutions containing bean extract and starch with or without amylase, respectively. A_C_ and A_D_ are the absorbances of mixed solutions containing starch and amylase or only starch, respectively. The inhibitory activity was expressed as the IC_50_ value.

#### α‐Glucosidase inhibition assay

The inhibition of α‐glucosidase activity was determined by measuring the amount of 4‐methylumbelliferone hydrolyzed from 4‐MUG (fluorogenic substrate) (Podsedek et al., 2014). For the α‐glucosidase assay, the fluorescence (A_1_) (excitation at 390 nm and emission at 410 nm) was measured using a Synergy H1 instrument (BioTek Instruments Inc.). An α‐glucosidase control (A_3_) sample containing buffer, substrate (4‐MUG), and enzyme, a blank (A_2_) containing bean extract, buffer, and enzyme, and a blank control (A_4_) containing the buffer and enzyme were prepared. All samples were assayed in triplicate, and acarbose was used as positive control. The rate of α‐glucosidase inhibition activity was calculated using the following equation:

(3) $$\text{inhibition}\left(\%\right)=\left[1-\frac{\left(A_{1}-A_{2}\right)}{\left(A_{3}-A_{4}\right)}\right]\times 100$$

where A_1_, A_2_, A_3_, and A_4_ are the values of fluorescence of the sample, blank, control, and blank control, respectively.

#### Lipase inhibition assay

The inhibition of lipase activity was determined by measuring the release of 4‐methylumbelliferone from 4‐MUO using a fluorimetric method (Cha, Song, Kim, & Pan, 2012). The fluorescence (A_a_) (excitation at 360 nm and emission at 460 nm) was measured using the Synergy H1 instruments. Orlistat was used as a positive control, and all samples were assayed in triplicate. The percentage of lipase inhibition activity was calculated by the following equation:

(4) $$\text{inhibition}\left(\%\right)=\left[1-\frac{\left(A_{a}-A_{b}\right)}{\left(A_{c}-A_{d}\right)}\right]\times 100,$$

where A_a_, A_c_, A_b_, and A_d_ are the values of fluorescence of the sample, control, blank, and blank control, respectively.

### Maillard reaction products (MRPs)

For the analysis of MRPs from the different samples, 2 g of dried coffee powder was mixed with 50 ml of distilled water. The mixture was heated at 100°C and stirred at 150 rpm (magnetic stirrer, iT‐0713; Shanghai Yiheng Scientific Instruments Co., Ltd., China) for 1 hr. The extract containing MRPs was filtered through a Whatman nylon membrane filter (0.45 μm) and stored at 4°C. The absorbances of the filtered extract at 294 and 420 nm were measured using a UV spectrophotometer (Ajandouz, Tchiakpe, Ore, Benajiba, & Puigserver, 2001). The absorbance ratio (A294/A420) was calculated to monitor the transformation of UV‐absorbing compounds into brown polymers. Each sample was determined in triplicate.

### In vitro digestion and absorption

#### In vitro simulation of digestion—potential bio‐accessibility (GE)

In vitro digestion was carried out as described by Minekus et al. (2014). The prepared samples which have simulated digestion process were incubated at 37°C for 120 min (37°C) in the dark for in vitro digestion. Thereafter, the samples were centrifuged at 16,099.2 *g* and the supernatants were collected for further analysis.

#### In vitro simulation of absorption—potential bioavailability (AE)

The solutions obtained after in vitro digestion were transferred to dialysis sacks (D9777–100FT, Sigma‐Aldrich), placed in a flask containing 50 ml of phosphate‐buffered saline, and incubated 2 hr in a magnetic stirrer (10 rpm, 37°C). The substances were passed through the membrane, treated as an equivalent of the raw material absorbed in the intestine after digestion (at least 75% efficiency). Each sample was determined in triplicate.

#### Theoretical calculations

To characterize the biologically active compounds in view of their GE and AE, the following factors were calculated (Gawlik‐Dziki et al., 2015). The relative phenolic bio‐accessibility index (PBC), which is an indication of potential bio‐accessibility of phenolic compounds, was calculated as follows:

(5) $$\text{PBC}=C_{\mathrm{GE}}/C_{\mathrm{BE}}$$

The relative phenolics bio availability index (RBV), which is an indication of potential bioavailability of phenolic compounds, was calculated as follows:

(6) $$\text{RBV}=C_{\mathrm{AE}}/C_{\mathrm{BE}}$$

The phenolics bioavailability index (PBV), which is an indication of the bioavailability of phenolic compounds, was calculated as follows:

(7) $$\text{PBV}=C_{\mathrm{AE}}/C_{\mathrm{GE}},$$

where C_BE_ is the phenolic content in raw extract (BE), C_GE_ is the phenolic content in extracts after simulated gastrointestinal digestion (GE), and C_AE_ is the phenolic content in extracts after simulated intestinal absorption (AE).

The antioxidant bioavailability index (ABV), which is an indication of the bio‐availability of antioxidative compounds, was calculated as follows:

(8) $$\text{ABV}=A_{\mathrm{GE}}/A_{\mathrm{AE}},$$

where A_GE_ is the activity of the extract after simulated gastrointestinal digestion (GE) and A_AE_ is the activity of extract after simulated intestinal absorption (AE).

### Statistical analysis

Experimental data were analyzed using Origin 8.0 (Microsoft Software, Inc., Northampton, USA) and SPSS 20.0 (Chicago, IL, USA). Differences between samples were identified using analysis of variance (ANOVA) and Duncan's multiple‐range test. A *p*‐value < 0.05 was considered significant.

## RESULTS AND DISCUSSION

### Energy consumption analysis of the drying procedures

Energy consumption is the main factor influencing the choice of a drying method for green coffee beans. Supporting Information Table S1 shows the energy consumption of the eight different drying procedures. MVD at 1.0 kW had the shortest drying time of 0.63 hr/kg, which increased by 211.11%, 369.84%, and 142.86% when the microwave power was set at 0.5 kW, 0.3 kW, and CMVD (1.0 + 0.5 + 0.3 kW), respectively. RTD had the longest drying time of 120 hr/kg, followed by HD‐40, HD‐50, and FD‐50, with 32.0, 18.0, and 7.1 hr/kg, respectively. In addition, the energy consumption of MVD beans at 1 kW was the minimum (1.88 kwh/kg), and energy consumption increased by 324.47%, 182.45%, 213.30%, 372.87%, and 143.62% under HD‐40, HD‐50, MVD‐0.5, MVD‐0.3, and CMVD, respectively. Meanwhile, FD‐50 was the most energy‐consuming (23.67 kwh/kg). Thus, MVD was the least time‐consuming and energy‐consuming approach when compared to the other drying procedures.

### Qualitative and quantitative analyses of phenolic acids

**Figure 1:**
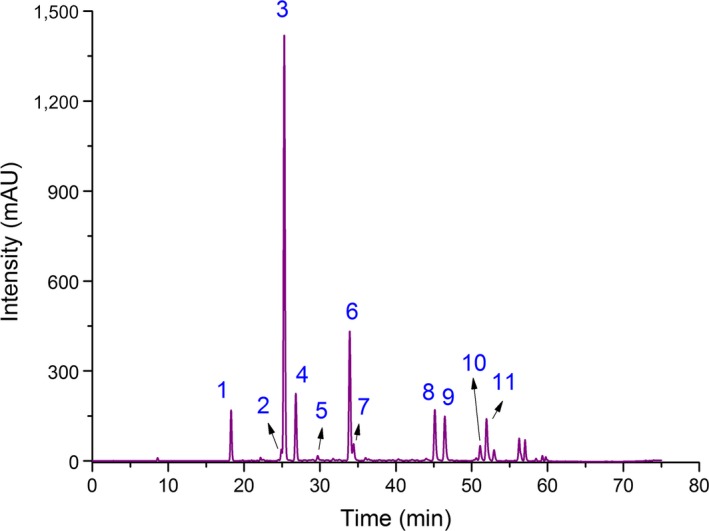
HPLC chromatogram of phenolic standards (330 nm): (1) 3‐caffeoylquinic acid, (2) 3‐feruloylquinic acid, (3) 5‐caffeoylquinic acid, (4) 4‐caffeoylquinic acid, (5) l‐epicatechin, (6) 5‐feruloylquinic acid, (7) 4‐feruloylquinic acid, (8) 3,4‐dicaffeoylquinic acid, (9) 3,5‐dicaffeoylquinic acid, (10) 1,3,5‐tricaffeoylquinic acid, and (11) 4,5‐dicaffeoylquinic acid

Eleven phenolic acids of green coffee beans processed by the different drying procedures were identified by HPLC‐DAD (Figure 1). Mills, Oruna‐Concha, Mottram, Gibson, and Spencer (2013). previously reported the same chromatographic peak sequence for eight phenolic acids. l‐EP was not detected in MVD samples, but all phenolic acids were detected in the other dried samples (Table 1). This phenomenon may be explained by the degradation of this compound at high temperatures. RTD beans had the highest content of l‐EP, which was similar to that in green coffee beans. However, 3‐FQA, 4‐FQA, and 5‐FQA contents of MVD beans were significantly higher than those in green, RTD, HD, and FD beans, while the highest content of these phenolic acids was found in MVD‐1.0 beans. Green coffee beans had the highest contents of 1,3,5‐triCQA, 3‐CQA, 3,4‐diCQA, and 3,5‐diCQA, and the lowest losses of these compounds were noted for MVD‐1.0, FD‐50, FD‐50, and MVD‐0.3, respectively. Additionally, FD beans had the highest contents of 4‐CQA and 4,5‐diCQA, while the content of 5‐CQA in MVD‐0.5 beans was higher than that in others. These results suggested that MVD is the most suitable method among the tested methods for the preservation of 3‐FQA, 4‐FQA, 5‐FQA, 1,3,5‐triCQA, 5‐CQA, and 3,5‐diCQA, while FD was more suitable for 3‐CQA, 3,4‐diCQA, 4‐CQA, and 4,5‐diCQA.

**Table 1:** Effect of drying procedures on the content of phenolic acids in coffee beans

Content (g/100 g DW)					
Phenolic acids	Green	RTD	HD‐40	HD‐50	FD‐50
L‐EP	0.14 ± 0.01^c^	0.16 ± 0.01^d^	0.07 ± 0.01^b^	0.07 ± 0.01^b^	0.06 ± 0.01^a^
3‐FQA	0.19 ± 0.01^c^	0.13 ± 0.01^a^	0.20 ± 0.01^d^	0.18 ± 0.01^bc^	0.18 ± 0.01^b^
4‐FQA	0.20 ± 0.01^c^	0.15 ± 0.01^a^	0.21 ± 0.01^b^	0.20 ± 0.01^c^	0.20 ± 0.02^d^
5‐FQA	1.13 ± 0.01^b^	0.81 ± 0.01^a^	1.33 ± 0.01 ^cd^	1.29 ± 0.02^c^	1.37 ± 0.03^d^
1,3,5‐triCQA	0.41 ± 0.01 ^g^	0.25 ± 0.01^a^	0.28 ± 0.01^b^	0.28 ± 0.01^c^	0.29 ± 0.01^d^
3‐CQA	0.50 ± 0.01 ^g^	0.29 ± 0.01^a^	0.38 ± 0.01^c^	0.37 ± 0.01^b^	0.46 ± 0.01^f^
4‐CQA	0.49 ± 0.01^de^	0.29 ± 0.01^a^	0.43 ± 0.01^c^	0.41 ± 0.01^b^	0.53 ± 0.01^f^
5‐CQA	0.83 ± 0.26^b^	0.55 ± 0.01^a^	1.16 ± 0.01 ^cd^	1.09 ± 0.02^c^	1.30 ± 0.01^def^
3,4‐diCQA	0.41 ± 0.01 ^g^	0.24 ± 0.01^a^	0.30 ± 0.01^b^	0.30 ± 0.01^b^	0.34 ± 0.01^f^
3,5‐diCQA	0.41 ± 0.01^f^	0.23 ± 0.01^a^	0.28 ± 0.01^b^	0.28 ± 0.01^b^	0.28 ± 0.01^c^
4,5‐diCQA	0.11 ± 0.01^b^	0.08 ± 0.01^a^	0.16 ± 0.01^e^	0.12 ± 0.01^c^	0.20 ± 0.01 ^g^

Content (g/100 g DW)				
Phenolic acids	MVD‐1.0	MVD‐0.5	MVD‐0.3	CMVD
L‐EP	ND	ND	ND	ND
3‐FQA	0.25 ± 0.01 ^g^	0.23 ± 0.01^f^	0.21 ± 0.01^e^	0.23 ± 0.01^f^
4‐FQA	0.25 ± 0.01^f^	0.23 ± 0.01^e^	0.22 ± 0.01^d^	0.23 ± 0.01^e^
5‐FQA	1.52 ± 0.01^f^	1.47 ± 0.02^e^	1.51 ± 0.01^ef^	1.50 ± 0.05^ef^
1,3,5‐triCQA	0.30 ± 0.01^f^	0.30 ± 0.01^e^	0.29 ± 0.01^d^	0.30 ± 0.01^e^
3‐CQA	0.44 ± 0.01^e^	0.42 ± 0.01^d^	0.44 ± 0.01^e^	0.38 ± 0.01^c^
4‐CQA	0.50 ± 0.01^e^	0.48 ± 0.01^d^	0.50 ± 0.01^e^	0.43 ± 0.02^c^
5‐CQA	1.28 ± 0.01^def^	1.39 ± 0.01^f^	1.33 ± 0.03^ef^	1.20 ± 0.03^cde^
3,4‐diCQA	0.33 ± 0.01^e^	0.32 ± 0.01^d^	0.34 ± 0.01^ef^	0.31 ± 0.01^c^
3,5‐diCQA	0.28 ± 0.01^c^	0.30 ± 0.01^e^	0.29 ± 0.01^d^	0.29 ± 0.01^d^
4,5‐diCQA	0.19 ± 0.01^f^	0.19 ± 0.01^f^	0.19 ± 0.01^f^	0.13 ± 0.01^d^

*Notes*

Results are expressed as mean ± standard error. Different capital letters in the same row indicate significant differences at *p* < 0.05. Undertake the above Table.

### Total phenolics, and their bio‐accessibility and bioavailability

**Figure 2:**
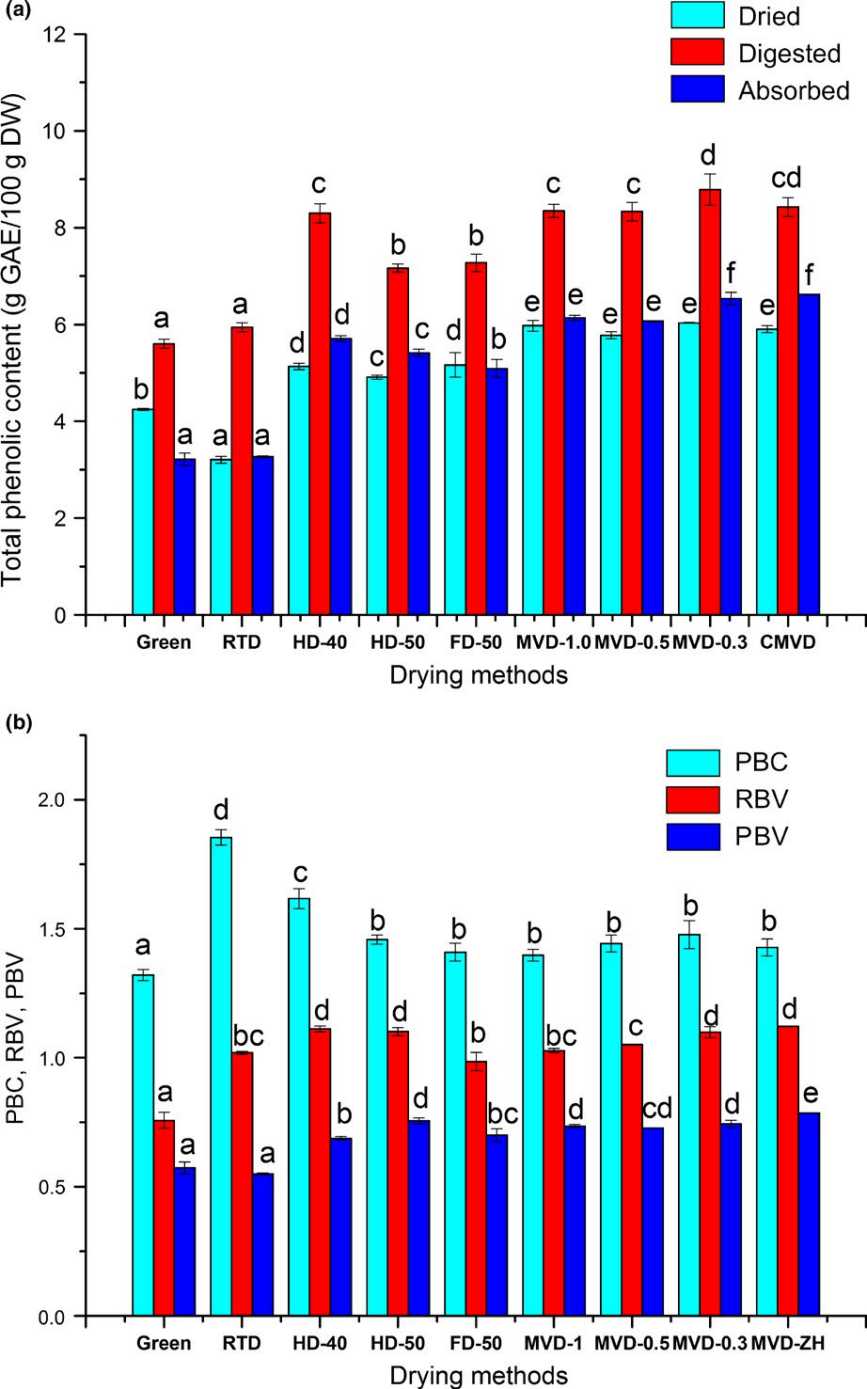
**Effect of different drying procedures on the total phenolic contents (a), potential bio‐accessibility, and bio‐availability (b) of green coffee beans**

Data on the TPC of green and dried coffee beans as well as their in vitro digestion and absorption are presented in Figure 2a. The TPC of green coffee beans was 4.24 ± 0.02 g GAE/100 g DW, and the TPCs in dried samples were significantly increased as compared to that in green coffee beans, except for RTD samples. The TPC in RTD beans was decreased by approximately 24.0%, while the TPCs in HD‐40, HD‐50, FD‐50, MVD‐1.0, MVD‐0.5, MVD‐0.3, and CMVD were significantly increased by approximately 21.0%, 16.0%, 22.0%, 41.0%, 36.0%, 42.0%, and 39.0%, respectively, when compared to that in green coffee beans. MVD‐1.0 induced the strongest increase in TPC, and MVD beans had higher TPC than other dried beans. The decrease in TPC in RTD beans may be due to the long drying time and the consequent degradation of phenolic. Chang et al. (2006). found that the TPC of tomatoes increased by 13%–29% after convective drying compared to raw samples. The increase in TPC in the dried coffee beans maybe owing to the release of polyphenolic compounds from the food matrix during drying. As for in vitro digestion and absorption, the TPC in all samples increased after in vitro digestion and decreased after in vitro absorption. Similarly, Dziki et al. (2015). reported that the TPC of dried coffee beans in five different areas increased after in vitro digestion and decreased after in vitro absorption.

The potential bioaccessibility and bioavailability indexes of coffee phenolic compounds are presented in Figure 2b. PBC and RBV were calculated to obtain the extraction efficiency in the simulated gastrointestinal tract. The PBC values all exceeded 1.0, which indicates a strong potential bio‐accessibility, especially in RTD beans (1.85 ± 0.03). However, the RBV values were close to 1.0, which indicates a common level of potential bioavailability. PBV indicates the potential bioavailability of compounds released during digestion in vitro, and the highest value was found in CMVD beans (0.79 ± 0.01). Thus, interestingly, the dried coffee beans not only had a relatively high content of phenolics, but also had high bioaccessibility and bio‐availability parameter values.

### Antioxidant activity of the potentially bio‐accessible and bioavailable fraction

Coffee has many health‐benefiting activities, mainly owing to its excellent antioxidant activity (AA). To obtain an in‐depth view of the AA of the dried coffee beans, three methods were used to evaluate the AA: DPPH, FRAP, and ABTS analyses. The bio‐accessibility and bioavailability in vitro of antioxidants from the dried coffee beans are shown in Table 2. For the DPPH assay after in vitro digestion, the lowest capacity was determined for coffee beans prepared by MVD‐ZH (EC_50_ = 4.09 ± 0.16 mg DW ml^−1^), while the highest capacity was determined for green coffee beans (EC_50_ = 0.62 ± 0.03 mg DW ml^−1^). The FRAP determined for extracts obtained after simulated digestion was relatively low in comparison with other activities, and it ranged between 4.22 and 20.22 mg DW ml^−1^. As for the ABTS assay after simulated digestion, the values were higher than those of other activities (1.59 to 5.13 mg DW ml^−1^).

**Table 2:** Bioaccessibility and bioavailability in vitro of antioxidants from coffee beans dried by the different procedures

Antioxidant Index	Drying Method	Bio‐accessibility in vitro EC_50_ (mg DW/mL)	Bio‐availability in vitro EC_50_ (mg DW/mL)	Bioavailability (ABV) factor
DPPH	Green	0.62 ± 0.03^a^	0.63 ± 0.01^a^	0.98
	RTD	2.23 ± 0.16^c^	1.42 ± 0.01^b^	1.57
	HD‐40	1.67 ± 0.16^b^	2.49 ± 0.01^c^	0.67
	HD‐50	2.33 ± 0.01^c^	2.89 ± 0.01^d^	0.81
	FD‐50	2.46 ± 0.11^c^	3.44 ± 0.01^e^	0.72
	MVD‐1.0	2.63 ± 0.11^c^	3.89 ± 0.01^f^	0.68
	MVD‐0.5	3.49 ± 0.32^d^	3.89 ± 0.08^f^	0.90
	MVD‐0.3	2.53 ± 0.29^c^	5.27 ± 0.05 ^h^	0.48
	MVD‐ZH	4.09 ± 0.16^e^	5.10 ± 0.01 ^g^	0.80
FRAP	Green	20.22 ± 0.09^e^	5.65 ± 0.08^abc^	3.58
	RTD	4.57 ± 0.09^bc^	5.57 ± 0.34^abc^	0.82
	HD‐40	4.22 ± 0.15^a^	5.42 ± 0.22^a^	0.78
	HD‐50	4.71 ± 0.08^c^	5.52 ± 0.04^ab^	0.85
	FD‐50	5.38 ± 0.11^d^	7.40 ± 0.06^e^	0.73
	MVD‐1.0	4.53 ± 0.03^bc^	6.57 ± 0.08^d^	0.69
	MVD‐0.5	4.40 ± 0.01^ab^	6.41 ± 0.13^d^	0.69
	MVD‐0.3	4.49 ± 0.06^b^	5.94 ± 0.16^c^	0.76
	MVD‐ZH	4.54 ± 0.02^bc^	5.84 ± 0.06^bc^	0.78
ABTS	Green	1.78 ± 0.06^a^	5.01 ± 0.85^bc^	0.36
	RTD	1.59 ± 0.25^a^	2.30 ± 0.01^a^	0.69
	HD‐40	2.42 ± 0.23^bc^	3.11 ± 0.25^a^	0.78
	HD‐50	2.11 ± 0.07^ab^	2.35 ± 0.19^a^	0.90
	FD‐50	2.53 ± 0.36^bc^	4.27 ± 0.89^b^	0.60
	MVD‐1.0	2.63 ± 0.15^bc^	3.04 ± 0.13^a^	0.86
	MVD‐0.5	5.13 ± 0.50^e^	5.76 ± 0.13^c^	0.89
	MVD‐0.3	3.03 ± 0.19 ^cd^	4.33 ± 0.33^b^	0.70
	MVD‐ZH	3.57 ± 0.16^d^	2.91 ± 0.23^a^	1.23

*Notes*

Results are expressed as mean ± standard error. Different capital letters in the same row indicate significant differences at *p* < 0.05.

Interestingly, the AA of extracts obtained after simulated absorption was significantly lower than that determined for samples obtained after simulated digestion. Similarly, Dziki et al. (2015) reported that the antiradical activity of simulated absorbed coffee beans was lower than that of simulated digested beans. The potential bioavailability of DPPH, FRAP, and ABTS AA activities ranged from 0.63 to 5.27 mg DW ml^−1^, 5.42 to 7.40 mg DW ml^−1^, and 2.30 to 5.76 mg DW ml^−1^, respectively. DPPH and ABTS radical scavenging activities of coffee beans increase with dehydration. Besides, the highest bioavailabilities of DPPH, FRAP, and ABTS scavenging activities were 1.57 (ABV), 3.58, and 1.23 for RTD, green, and MVD‐ZH beans, respectively.

### Changes in PPO activity

**Figure 3:**
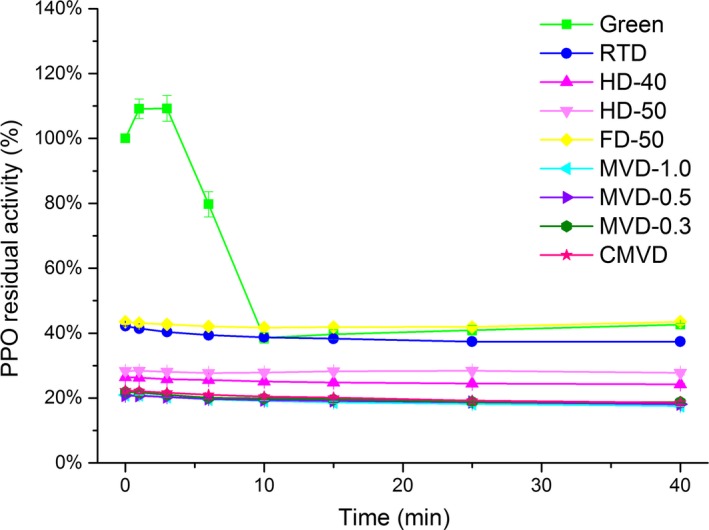
**Effect of different drying procedures on the residual PPO activity (%) of green coffee beans**

During dehydration procedures, oxidative and hydrolytic enzymes are inactivated (Benlloch‐Tinoco, Igual, Rodrigo, & Martínez‐Navarrete, 2013), which may explain the increase in AA we observed. The percentage residual activity of PPO in green and dried coffee beans is shown in Figure 3. FD‐50 beans had the highest enzyme activity, followed in order by green > FD > HD > MVD. The enzyme activities of dried coffee beans were slightly decreased, and the greatest changes in enzyme activity in green coffee beans were observed at 0–10 min. Thus, drying reduced the PPO activity in coffee beans, and MVD was found to the best method to deactivate PPO and obtain the highest antioxidant activity.

### Inhibition of LPO and TFC

As coffee powders have a strong ability to inhibit LPO, the inhibition of LPO in green and dried coffee beans was compared in this study (Supporting Information Figure S1). Green coffee beans had a lowest inhibitory activity (41.42% ± 1.66%), whereas the drying process led to an increase in the inhibitory activity. Beans dried under MVD‐1.0 had the strongest inhibitory activity (106.5% ± 2.6%), and compared to the green coffee beans, the LPO inhibition was increased by 94.8%, 82.6%, 66.5%, 21.0%, 46.4%, 78.2%, and 57.1% in RTD, HD‐40, HD‐50, FD‐50, MVD‐0.5, MVD‐0.3, and CMVD samples, respectively. Coffee powders have been added to bread to increase the inhibition of LPA. Świeca et al. (2017) reported that the inhibition of LPO in bread supplemented with coffee powder was higher than that in control bread.

The TFCs of green and dried coffee beans are shown in Supporting Information Figure S2. The TFC of green coffee beans was 10.42 ± 0.14 g rutin/100 g DW, and TFC significantly increased as compared to that in green coffee beans in dried samples, except RTD samples. This tendency was in accordance with that of TPC. The TFC in RTD beans was decreased by approximately 35.0%, whereas in HD‐40, HD‐50, FD‐50, MVD‐1.0, MVD‐0.5, MVD‐0.3, and CMVD samples, TFC significantly increased by 12.0%, 5.0%, 12.0%, 34.0%, 29.0%, 35.0%, and 28.0%, respectively, when compared to that in green coffee beans. TFC increased the most in MVD‐0.3, and MVD beans had higher TPCs than other dried beans.

### Enzyme inhibitory activities

In the human body, pancreatic α‐amylase and intestinal α‐glucosidase hydrolyze dietary carbohydrates and decompose oligosaccharides and disaccharides into monosaccharides, which can be absorbed (Sales et al., 2012). However, the inhibition of these enzymes is important for the treatment of noninsulin‐dependent diabetes, because it will slow down the release of glucose in the blood. Significant differences (*p* < 0.05) in α‐amylase and α‐glucosidase inhibitory activities were observed among the dried coffee beans (Table 3). The inhibition of α‐amylase (IC_50_) ranged from 47.58 ± 0.36 to 198.92 ± 6.82 mg DW ml^−1^. FD beans showed the lowest inhibitory activity, while RTD beans presented the highest inhibitory activity. The inhibition of α‐glucosidase was weaker than that of α‐amylase. The inhibition of α‐glucosidase (IC_50_) ranged from 28.75 ± 1.27 to 54.62 ± 5.21 mg DW ml^−1^. MVD‐1.0 samples had the strongest inhibitory activity, and RTD samples had the lowest inhibitory activity.

**Table 3:** α‐Amylase, α‐glucosidase, and pancreatic lipase inhibitory activities of coffee beans dried by the different procedures

IC_50_ (mg DW/mL)			
Drying method	α‐amylase	α‐glucosidase	Pancreatic lipase
RTD	47.58 ± 0.36^a^	54.62 ± 5.21^c^	53.01 ± 0.33^b^
HD‐40	60.11 ± 0.05^b^	35.63 ± 0.30^b^	53.03 ± 0.49^b^
HD‐50	60.11 ± 0.70^b^	33.97 ± 1.13^ab^	55.28 ± 0.12^c^
FD‐50	198.92 ± 6.82^e^	32.36 ± 0.35^ab^	53.61 ± 0.16^b^
MVD‐1.0	71.34 ± 1.77^c^	28.75 ± 1.27^a^	54.86 ± 0.73^c^
MVD‐0.5	86.96 ± 0.81^d^	33.83 ± 0.27^ab^	52.00 ± 0.21^a^
MVD‐0.3	89.54 ± 1.47^d^	34.59 ± 3.56^ab^	51.68 ± 0.07^a^
CMVD	51.61 ± 0.95^a^	34.45 ± 0.83^ab^	51.49 ± 0.29^a^

*Notes*

Results are expressed as mean ± standard error. Different capital letters in the same row indicate significant differences at *p* < 0.05.

Pancreatic lipase, another important enzyme in the body, is responsible for the digestion of dietary fat and therefore can play a role in the prevention and cure of overweight and obesity (Podsedek et al., 2014). The pancreatic lipase inhibitory activities of the green and dried coffee beans are presented in Table 3. The IC_50_ values varied from 51.49 ± 0.29 to 55.28 ± 0.12 mg DW ml^−1^, and no significant difference was found. This finding indicated that drying has no significant effect on the inhibition of pancreatic lipase.

### MRPs

Data on UV absorbance (A_294_), browning intensity (A_420_), and the transformation of UV‐absorbing substances into brown polymers (A_294_/A_420_) are shown in Supporting Information Table S2. The formation of intermediate compounds of MRPs was expressed as A_294_ and A_420_, which were used to determine their intensity (Ajandouz et al., 2001). The values of UV absorbance, browning intensity, and the transformation ranged from 1.95 ± 0.16 to 2.66 ± 0.14, 0.36 ± 0.01 to 1.37 ± 0.24, and 1.94 ± 0.10 to 5.97 ± 0.41, respectively. The values of A_294_/A_420_ increased in the order: RTD < HD‐40 < HD‐50 < FD‐50 < MVD, which may be explained by the increase in drying temperature, which is in agreement with previous reports (Benzing‐Purdie, Ripmeester, & Ratcliffe, 1985). MRPs have been reported to have numerous biological activities, including antioxidant activities and metal‐chelating property (Lertittikul, Benjakul, & Tanaka, 2007).

### Pearson correlation coefficient analysis

The Pearson correlation coefficients for the different chemical properties of the dried coffee beans are listed in Supporting Information Table S3. The eight chemical indexes analyzed included TPC, TFC, DPPH, FRAP, and ABTS, and inhibition of α‐amylase, α‐glucosidase, and pancreatic lipase. TPC was significantly positively correlated with TFC, DPPH, and FRAP, and negatively with the inhibition of α‐amylase. Accordingly, Méndez‐Lagunas, Rodríguez‐Ramírez, Cruz‐Gracida, Sandoval‐Torres, & Barriada‐Bernal, 2017 reported a significant positive correlation between TPC and antioxidant activity. Similar correlations were observed between TFC, and DPPH and FRAP. Interestingly, FRAP was significantly negatively correlated with the inhibition of α‐glucosidase. Apostolidis, Kwon, and Shetty (2007) reported increased α‐glucosidase inhibitory activity in Roquefort cheese, which could be attributed to specific phenolics or secondary metabolites produced by *Penicillium* species. Vadivel and Biesalski (2011) reported that oil‐fried, sprouted seeds had higher TPC and weaker α‐glucosidase inhibitory activity than raw seeds and traditionally processed seeds. These findings are in agreement with ours.

## CONCLUSIONS

In the present study, MVD was the least time‐consuming and energy‐consuming of all drying processes tested. Moreover, among 11 phenolic acids evaluated, MVD beans had the highest contents of 5‐CQA, 3‐FQA, 4‐FQA, 5‐FQA, 3,5‐diCQA, and 1,3,5‐triCQA when compared to beans dried by the other methods. Furthermore, MVD beans had the highest values of TPC, TFC, and AA as compared to green and other dried beans, and MVD induced the strongest inhibition of enzyme activities (α‐amylase, α‐glucosidase, and pancreatic lipase) and LPO when compared to the other drying methods. Interestingly, TPC and TFC showed a significant, positive correlation with DPPH and FRAP. The inhibitory activity of α‐glucosidase was negatively correlated with TPC and TFC, which indicated that TPC and TFC increase along with α‐glucosidase activity. Thus, MVD is suggested to be the most suitable drying technique for the preservation of phenolic compounds and bioactivities of green coffee beans in the industry and encouraged its application and development of similar methods with other similar agricultural products.

## CONFLICT OF INTEREST

The authors declare that they do not have any conflict of interest.

## ETHICAL STATEMENTS

This study does not involve any human or animal testing.
