# Supplementary material for: Evaluation of the impact of different drying methods on the phenolic compounds, antioxidant activity, and in vitro digestion of green coffee beans
Source: Food Sci Nutr. 2019 Feb 11;7(3):1084–95. doi: 10.1002/fsn3.948 (PMC6418437; doi:10.1002/fsn3.948)

**Supplementary material**

Supplemental Table 1. Energy consumption of the different drying processes.

______________________________________________________________________________

Drying method Drying time (h/kg) Energy consumption (kwh/kg)

______________________________________________________________________________

RTD 120.00 ND

HD-40 32.00 6.10

HD-50 18.00 3.43

FD-50 7.10 23.67

MVD-1.0 0.63 1.88

MVD-0.5 1.33 4.01

MVD-0.3 2.33 7.01

CMVD 0.90 2.70

_______________________________________________________________________________

Supplemental Table 2. Maillard reaction products.

**_____________________________________________________________________________**

Absorbance (nm) 294 nm 420 nm (294 nm/420 nm)

_____________________________________________________________________________

RTD 2.66 ± 0.14 1.37 ± 0.24 1.94 ± 0.10

HD-40 2.47 ± 0.29 0.64 ± 0.28 3.89 ± 0.41

HD-50 2.44 ± 0.10 0.61 ± 0.01 3.97 ± 0.15

FD-50 2.28 ± 0.15 0.54 ± 0.01 4.24 ± 0.23

MVD-1.0 2.34 ± 0.12 0.52 ± 0.22 4.53 ± 0.25

MVD-0.5 2.43 ± 0.23 0.45 ± 0.01 5.43 ± 0.40

MVD-0.3 2.15 ± 0.19 0.36 ± 0.01 5.97 ± 0.41

CMVD 1.95 ± 0.16 0.36 ± 0.01 5.46 ± 0.39

_____________________________________________________________________________

Supplemental Table 3. The Person correlation coefficients for different chemical properties of different dried coffee beans.

_____________________________________________________________________________________

TPC TFC DPPH FRAP ABTS

_____________________________________________________________________________________

TPC 1.000

TFC 0.998** 1.000

DPPH 0.920** 0.913** 1.000

FRAP 0.983** 0.979** 0.947** 1.000

ABTS 0.632 0.649 0.589 0.673 1.000

α-amylase 0.158 0.158 0.053 0.250 0.139

α-glucosidase -0.889** -0.886** -0.666 -0.841** -0.475

Pancreatic lipase -0.224 -0.210 -0.463 -0.311 -0.391

_____________________________________________________________________________________

_____________________________________________________________________________________

α-amylase α-glucosidase Pancreatic lipase

_____________________________________________________________________________________

TPC

TFC

DPPH

FRAP

ABTS

α-amylase 1.000

α-glucosidase -0.339 1.000

Pancreatic lipase 0.062 -0.179 1.000

_____________________________________________________________________________________

Undertake the above Table.

***p* < 0.01.

Supplemental Figure 1. Linoleic acid peroxidation-inhibitory activity of green beans and beans dried by the different approaches.


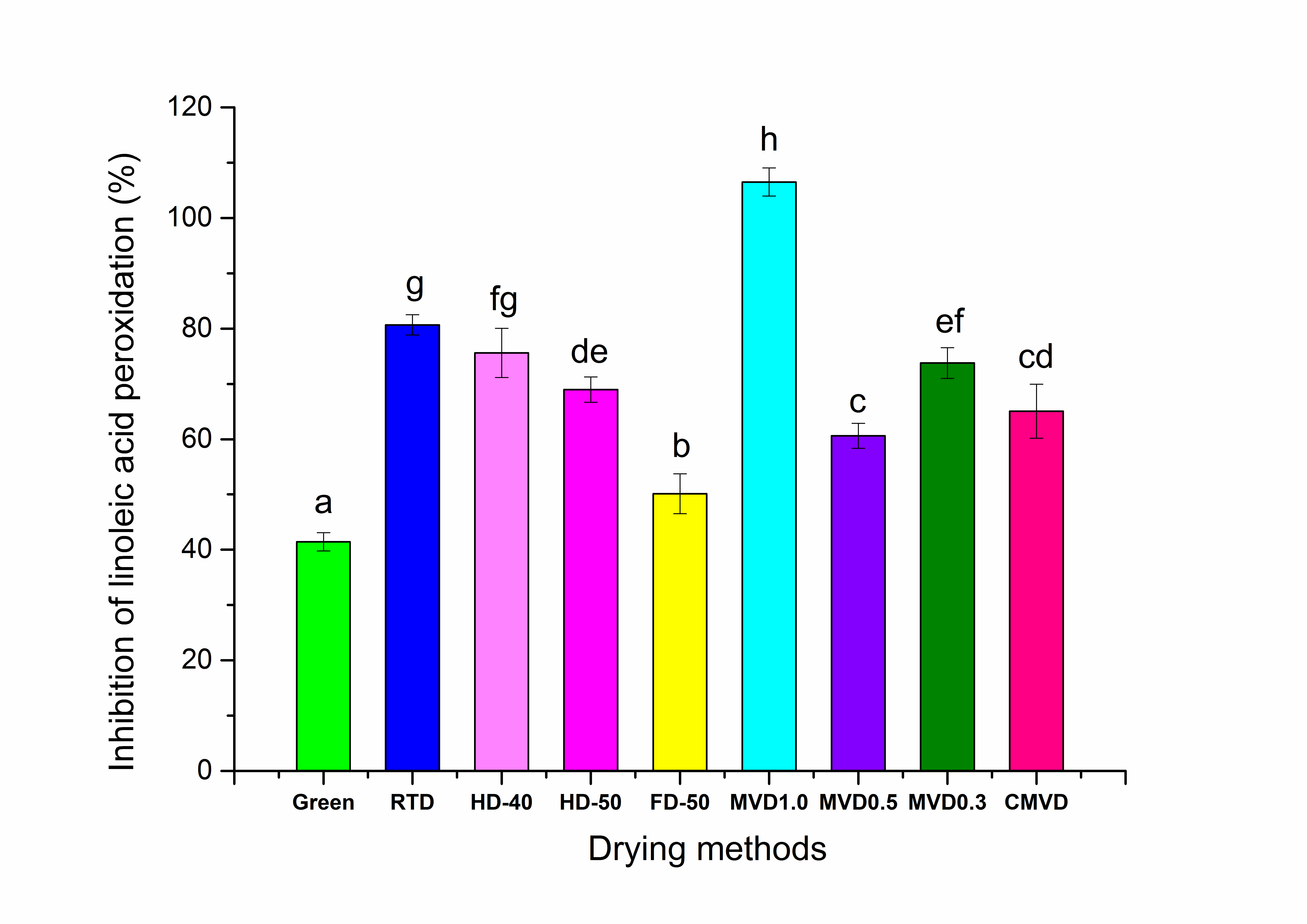


Supplemental Figure 2. Effect of different drying processes on the total flavonoid content of coffee beans.


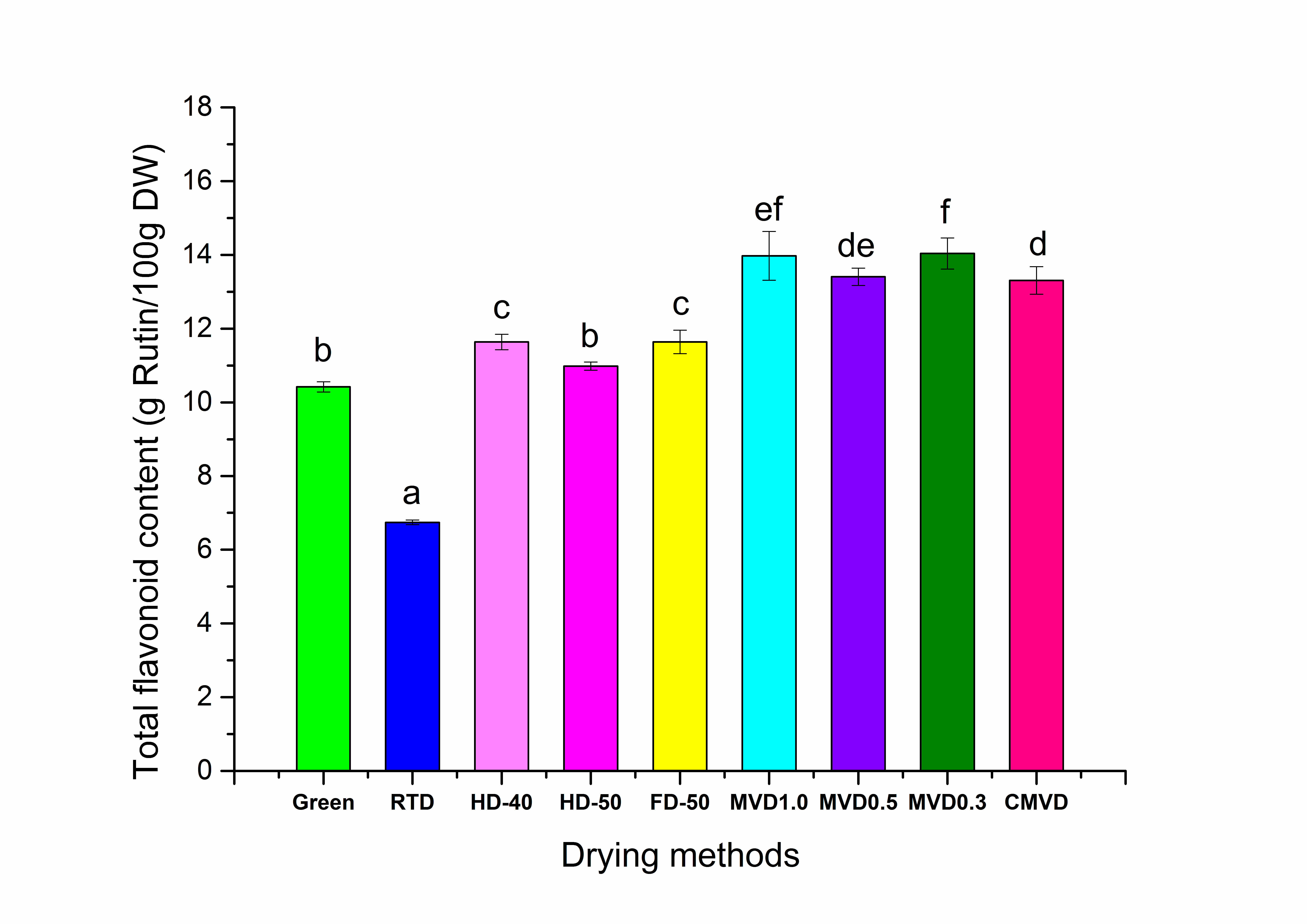

Supplement: Supplementary file 1 [file FSN3-7-1084-s001.doc]
